# Oneiroid catatonia due to the usage of spice: The case study

**DOI:** 10.1192/j.eurpsy.2021.2167

**Published:** 2021-08-13

**Authors:** O. Pityk, A. Pushko, N. Striltsiv, O. Lutska, N. Karbovskyi

**Affiliations:** 1 Psychiatry, Narcology And Medical Psychology Department, Ivano-Frankivsk National Medical University, Ivano-Frankivsk, Ukraine; 2 Medical Director, Precarpathian Regional Clinical Center of Ivano-Frankivsk Regional Council, Ivano-Frankivsk, Ukraine; 3 Department Of The First Psychotic Episode And Crisis Conditions, Precarpathian Regional Clinical Center of Ivano-Frankivsk Regional Council, Ivano-Frankivsk, Ukraine; 4 Student, Ivano-Frankivsk National Medical University, Ivano-Frankivsk, Ukraine

**Keywords:** Designer drugs, oneiroid catatonia, synthetic cannabinoids

## Abstract

**Introduction:**

Designer drugs, as a term, first came about in the 1980s. Most of these “designer drugs” have synthetic cannabinoids and other psychoactive formulas difficulty to detect.

**Objectives:**

A 28 year man was referred to the hospital.

**Methods:**

CT brain and EEG were also normal.

**Results:**

Among 7 days before attending the hospital the patient had a strange behaviour. He was staying like in changed reality. The day before admission he got irritable in the evening was reporting that he could hear animal’s imperative voices “we together with squirrel, dolphin visited giraffe, that someone told to jump from the window”. That symptoms were temporary after that he was shocked when realized that he was in a room. The patient has the history of marihuana use in the past 5 years, periodically. There is no evidence data about the usage of other narcotic substances. On examination he was alert, sitting on a same place looking at one point, sometimes trying to find something or suddenly standing and trying to go somewhere. He has a change of catatonic stupor and excitement. The psychomotor activity was changeable. While observing the patient during few days several times he disrobed all his clothes, staying or laying on a bed or suddenly freezing in one pose.

**Conclusions:**

Taking into account clinical symptoms, the patient developed, the conclusion was made about connection of patients’ oneiroid catatonia with the usage of “Spice” or “Designer drug”. Thus, designer drugs may sound like a safer alternative, but often can lead to serious mental disturbances.

**Disclosure:**

No significant relationships.

